# Correlation of TSH Levels with Inflammatory Markers in COVID-19 Patients: A Retrospective Study

**DOI:** 10.3390/jcm14238378

**Published:** 2025-11-26

**Authors:** Bartosz Krajewski, Martyna Kamińska, Jakub Ligęzka, Mateusz Romek, Agnieszka Żak-Gołąb, Michał Holecki

**Affiliations:** 1 Student Scientific Society at the Department of Internal Medicine, Autoimmunological Diseases and Diabetology, Medical University of Silesia, 40-752 Katowice, Poland; s82856@365.sum.edu.pl (M.K.);; 2 Faculty of Management, AGH University of Krakow, 30-059 Krakow, Poland; 3 Department of Internal Medicine, Autoimmunological Diseases and Diabetology, Medical University of Silesia, 40-752 Katowice, Poland

**Keywords:** Coronavirus disease 2019 (COVID-19), lymphocyte count, neutrophil-to-lymphocyte ratio, platelet count, thyroid-stimulating hormone

## Abstract

**Background:** COVID-19 caused by SARS-CoV-2 is an acute disease which may lead to severe systemic inflammation, causing multi-organ dysfunction and death. Studies indicated that thyroid-stimulating hormone (TSH) levels were lower when the infection was more severe. **Methods:** We conducted a retrospective study of 105 patients admitted from 2020 to 2023 to the University Clinical Centre in Katowice with a positive COVID-19 test. TSH levels, white blood cell count (WBC), platelet count (PLT), C-reactive protein (CRP), D-dimers, procalcitonin levels, lymphocyte count and percentage, and neutrophil-to-lymphocyte ratio (NLR) were evaluated. **Results:** The average age was 69.49 (SD 14.14) and the range was 36–95 years. A total of 53.2% of the population were male. After statistical analysis, lymphocyte count (*p* = 0.0038) correlated positively and NLR (*p* = 0.04682) correlated negatively with TSH level in COVID-19 patients, and PLT correlated positively with TSH level in the female population (*p* = 0.0384), while CRP (*p* = 0.81320), D-dimers (*p* = 0.974), WBC (*p* = 0.6862), Lymphocyte percentage (*p* = 0.1838), and procalcitonin (*p* = 0.906) did not reach statistical significance. **Conclusions:** TSH levels may be associated with lymphocyte count and NLR in patients with confirmed cases of SARS-CoV-2 infection, and with PLT in the female subgroup. Other evaluated inflammatory markers were not significant. These findings suggest that TSH has potential as a biomarker of disease severity, but further studies are needed to investigate this claim, and consideration should be given to other indicators of inflammation.

## 1. Introduction

Since the outbreak of the Coronavirus disease (COVID-19) pandemic, a total of 778,417,964 cases had been reported worldwide by 30 July 2025 [1]. The most common symptoms were respiratory in nature, such as pneumonia and acute respiratory distress syndrome (ARDS). However, the virus has also been associated with multi-organ failure, including acute kidney injury and cardiovascular complications [2]. Throughout the pandemic, an increasing number of potential non-respiratory complications have been linked to the infection. This has prompted investigations into other potential consequences of the infection, including its impact on the endocrine system.

Thyroid dysfunction has been repeatedly reported in COVID-19 patients [3]. Recent evidence indicates that COVID-19 infection can significantly affect thyroid function, leading to a spectrum of disorders. According to Anbardar et al. (2025) [4], thyroid dysfunction occurred in approximately 15% of patients with COVID-19, with prevalence depending on the disease severity, where 20.8% of severe cases had thyroid dysfunction and only 6.2% had mild cases. The most frequent abnormality was non-thyroidal illness syndrome (NTIS), observed in 5–58% of cases. Other thyroid dysfunctions were thyroid autoimmunity, thyroiditis, subclinical hypothyroidism, hypothyroidism, subclinical hyperthyroidism, and hyperthyroidism. The prevalence of these disorders varied, depending on the country.

Additionally, Graves’ disease incidence has increased during the pandemic, especially in the female population, whereas autoimmune thyroiditis has been reported more frequently in post-COVID survivors. Although many of these thyroid disturbances do not persist, chronic cases have also been described, especially in patients with severe disease or pre-existing autoimmune predisposition. These findings increase the importance of thyroid evaluation in COVID-19 patients but also of the post-infection follow-up [4].

**Figure 1:**
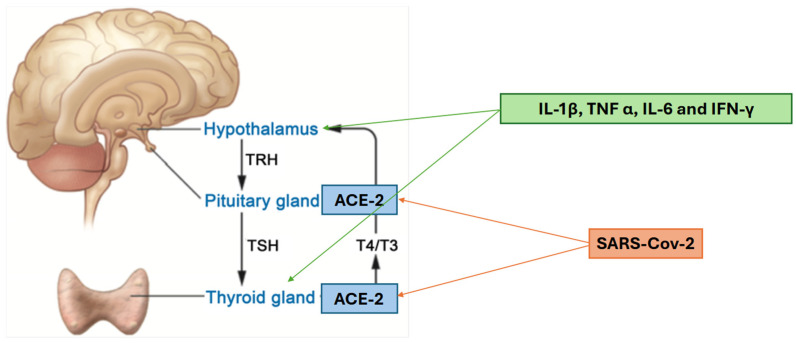
Influence of COVID-19 on HPT axis [6].

Of the various hypotheses regarding the pathomechanisms of thyroid dysregulation through SARS-CoV-2 infection, the most evidence-based are as follows. One possible mechanism involves angiotensin-converting enzyme 2 (ACE2), which is expressed in various human tissues, including the thyroid and pituitary glands. This expression suggests a potential link to the hypothalamic–pituitary–thyroid (HPT) axis. Importantly, ACE2 also serves as the entry receptor for SARS-CoV-2. Another proposed mechanism is the host’s immune response, resulting in the increase in cytokines such as interleukin-1β (IL-1β), tumour necrosis factor-α (TNF-α), interleukin-6 (IL-6), and interferon-γ (IFN-γ). These cytokines have direct impact on the hypothalamus and thyroid gland, potentially leading to the suppression of the HPT axis [5]. Those mechanisms are illustrated in Figure 1.

In addition to these two mechanisms, there is supporting evidence from clinical and pathological observations of a direct effect of SARS-CoV-2 on the thyroid gland. Autopsy studies have revealed structural changes in thyrocytes that suggest viral damage, and cases of subacute thyroiditis following a SARS-CoV-2 infection have been reported [7]. The above explanations of possible mechanisms support the fact that thyroid dysfunction occurs more frequently in patients infected with SARS-CoV-2.

Thyroid function differs among hospitalised patients based on their clinical condition. Euthyroid sick syndrome (ESS) or NTIS, characterised by decreased levels of free triiodothyronine (fT3) and/or free thyroxine (fT4) without an accompanying increase in thyroid-stimulating hormone (TSH), has been associated with disease severity [4,8,9]. As mentioned above, after the COVID-19 pandemic, the prevalence of autoimmunological thyroid disorders has increased. The mechanisms of new-onset thyroidal autoimmunity are molecular mimicry (due to similarities between spike protein, the nucleoprotein, and the membrane protein with thyroid peroxidase), neutrophil extracellular traps (similarly as in systemic lupus erythematosus, rheumatoid arthritis, myositis, and multiple sclerosis), and transcriptional changes in the immune genes (probably due to strong activation of interferon alpha and gamma pathways) [9].

Additionally, TSH levels themselves have been linked to the severity of the disease. Through metabolic and inflammatory pathways triggered by SARS-CoV-2, patients often present with lymphopenia, which correlates with disease severity and TSH levels. This reduction in lymphocyte count is believed to be the result of a combination of direct viral effects, cytokine-mediated damage, and metabolic stress [10]. Furthermore, inflammatory markers, including C-reactive protein (CRP), have been shown to correlate with TSH and fT3 levels [11]. Thyroid function was reported to be dysregulated in 61.1% of patients after mild or moderate SARS-CoV-2 infection, but only in 33.6% three months post infection. TSH levels were higher 2 months after the infection, compared to the follow-up 3 months after the infection. The risk of thyroid dysfunction’s persistence was higher in moderate cases rather than mild [12]. Some studies suggest the persistence of thyroid dysfunction 6 months after the infection, probably due to autoimmunity [13].

In light of the accumulating evidence on thyroid dysfunction in patients with SARS-CoV-2 infection, and the evidence-based links between the inflammatory response and the direct impact of the virus on the hypothalamic–pituitary–thyroid (HPT) axis, we performed a retrospective study involving 105 patients with confirmed SARS-CoV-2 infection. The aim of this study was to investigate this knowledge in clinical circumstances. While previous studies have indicated an association between thyroid dysfunction, systemic inflammation, and the severity of SARS-CoV-2 infection, the relationship between TSH and inflammatory markers remains unclear. Clarifying this association could provide new insights into the prognostic role of the HPT axis in the clinical course of the disease. Furthermore, investigating the inflammatory markers and their links to other systems, especially the endocrine system, could help clarify the overall design of the human inflammatory response.

## 2. Materials and Methods

### 2.1. Study Design and Population

A retrospective analysis was conducted on all patients admitted to the University Clinical Centre in Katowice between March 2020 and December 2023. Data were sourced from electronic medical records and anonymised prior to analysis. Patients who met the following inclusion criteria were enrolled:

- Confirmed SARS-CoV-2 infection (positive PCR test).
- Assessment of the following laboratory parameters within three days of confirming the SARS-CoV-2 infection: thyroid-stimulating hormone (TSH), platelet count (PLT), C-reactive protein (CRP), white blood cell count (WBC), D-dimer, procalcitonin, lymphocyte count, lymphocyte percentage, and neutrophil-to-lymphocyte ratio (NLR).

Patients without a TSH measurement during hospitalisation, with a previous history of thyroid dysfunction, and those with other autoimmune diseases (such as ulcerative colitis, asthma, and diabetes mellitus type 1) were excluded from the study. The division of those diseases is shown in Table 1.

**Figure 2:**
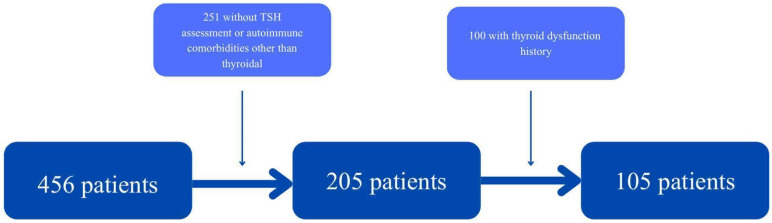
Patient’s exclusion process.

A total of 105 patients (49 women and 56 men) aged between 25 and 95 were included in this study. The exclusion process is illustrated in Figure 2.

TSH was considered the primary outcome and all other laboratory parameters were assessed in relation to its level. Statistical analysis was performed using the obtained parameters. It is important to note that the number of obtained records differed among selected parameters as shown in Table 2.

### 2.2. Laboratory Assessments

The presence of SARS-CoV-2 virus was confirmed through RT-PCR tests (MediPAN 2G + FAST COVID Kit [Medicofarma, Radom, Poland], 2019-Novel-Coronavirus [2019-nCOV], Triplex RT-qPCR Detection Kit [Vazyme, Nanjing, China], MutaPLEX Coronavirus Real-Time-RT-PCR-Kit [Immunodiagnostic], and Xpert Xpress SARS-CoV-2 and Xpert Xpress SARS-CoV-2/FLU/RSV [GeneXpert, Sunnyvale, CA, USA]).

Thyroid-stimulating hormone (TSH), procalcitonin, and D-dimer levels were measured using chemiluminescent immunoassay on the Roche Cobas Pro e801 analyzer [Roche Diagnostics, Mannheim, Germany] according to the manufacturer’s instructions.

Complete blood count (CBC) was performed using the Sysmex XN-1000 analyzer [Sysmex, Ono, Japan], which provides a standard five-part differential method and automated blood smear analysis with reagents provided by Sysmex.

C-reactive protein (CRP) was measured using the immunoturbidimetric method on a Roche Cobas Pro c503 analyser [Roche Diagnostics, Mannheim, Germany].

The neutrophil-to-lymphocyte ratio (NLR) was calculated using a standard formula.

### 2.3. Statistical Analysis

All analyses were performed using RStudio (version 2024.09.1). In all analyses, a *p* value of less than 0.05 was considered statistically significant. Outliers were identified and excluded using the Interquartile Range Method (IQR).

The normality of the distribution was assessed using the Shapiro–Wilk test. As there was an abnormal distribution for each parameter, statistical analysis was performed using Spearman’s rank correlation for the entire sample, as well as for males and females separately.

Due to a lack of significant correlation, CRP and WBC were each stratified into three groups and a Kruskal–Wallis test was applied (Table 3). This approach enabled potential differences between the groups to be identified that might not have been apparent when analysing the data as a continuous variable.

D-dimers, platelets (PLT), and procalcitonin were divided into two groups (see Table 4) and assessed using the Mann–Whitney U test, which revealed differences that would not have been apparent if the data had been treated as continuous.

Furthermore, lymphocyte count was evaluated using quantile regression. This approach enabled a more detailed analysis of the relationship with TSH at different quantile levels.

## 3. Results

**Figure 3:**
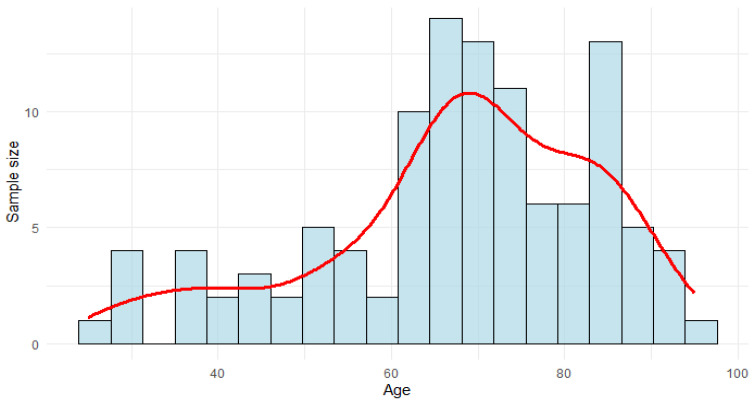
Age distribution among patients.

The average age of the population studied was 67.63 years (SD 16.26), ranging from 25 to 95 years (see Figure 3). A total of 52.7% of the population were male.

**Figure 4:**
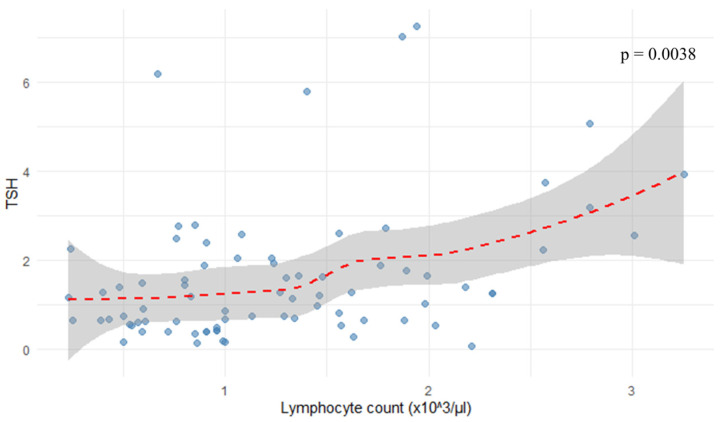
Correlation between lymphocyte count and TSH.

As shown in Figure 4, the analysis revealed a positive correlation between TSH levels and lymphocyte count (r = 0.324, *p* = 0.0038).

**Figure 5:**
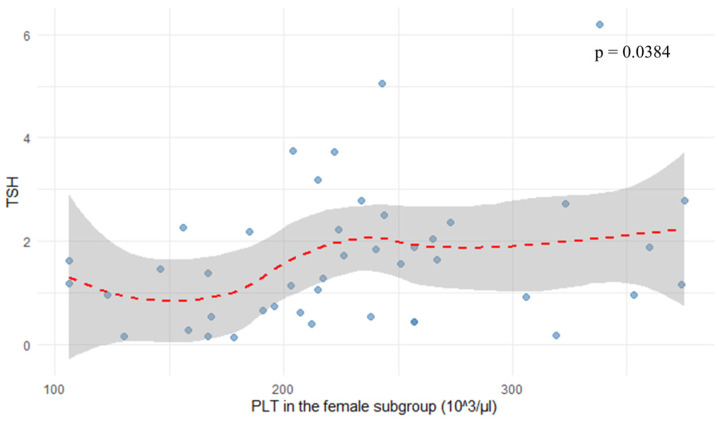
Correlation between platelet count and TSH in the female subgroup.

**Figure 6:**
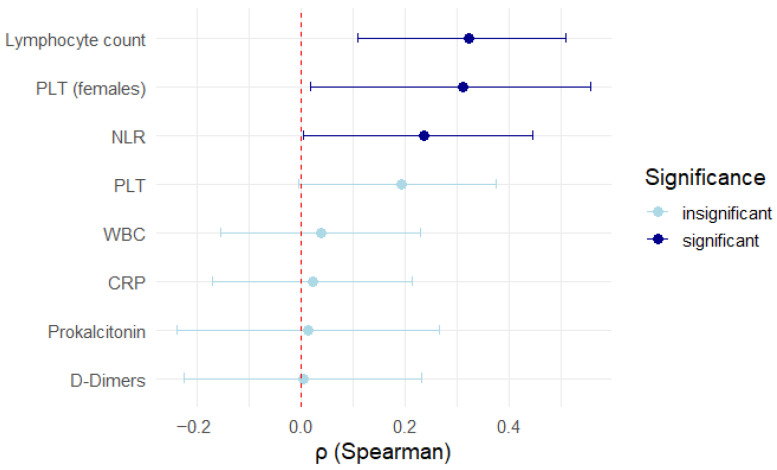
Results of the correlation of inflammatory markers with TSH.

**Figure 7:**
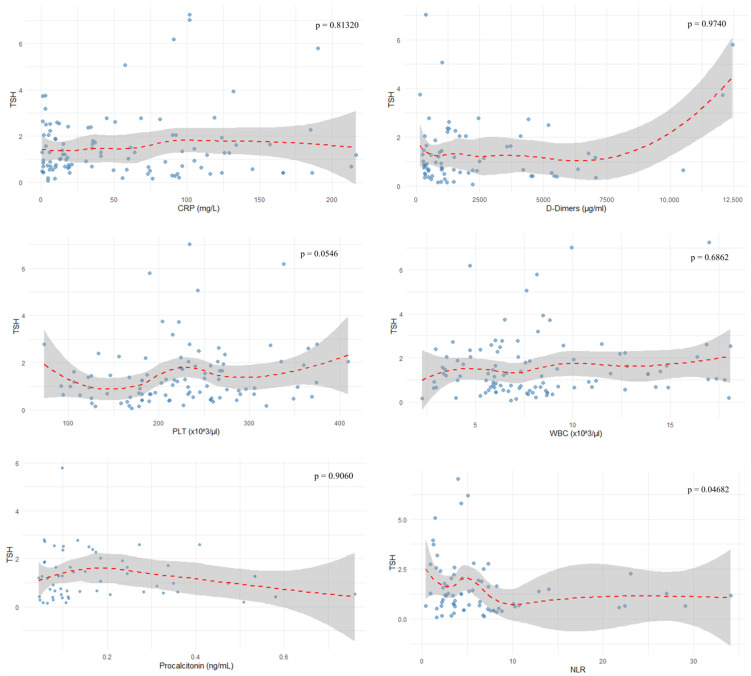
Correlations of other inflammatory markers with TSH.

In the female subgroup, platelet count (PLT) correlated positively with TSH levels (r = 0.313, *p* = 0.0384), as shown in Figure 5. Neutrophil-to-lymphocyte ratio showed a negative correlation with TSH (r = −0.237, *p* = 0.04682), as depicted in Figure 6 and Figure 7.

The evaluated parameters such as CRP (r = −0.023, *p* = 0.81320), D-dimers (r = 0.004, *p* = 0.9740), WBC (r = 0.04, *p* = 0.6862), lymphocyte percentage (r = 0.153, *p* = 0.1838), PLT (r = 0.193, *p* = 0.0546), and procalcitonin (r = 0.015, *p* = 0.9060) showed no correlation with TSH levels, as depicted in Figure 6. In these cases, the power analysis of the tests showed that they were too low (<10%) to reliably detect the effect. Therefore, the lack of statistical significance cannot be excluded; it can only be concluded that the available sample was not sufficient to detect it. The results of the statistical tests performed are outlined in Figure 7.

**Figure 8:**
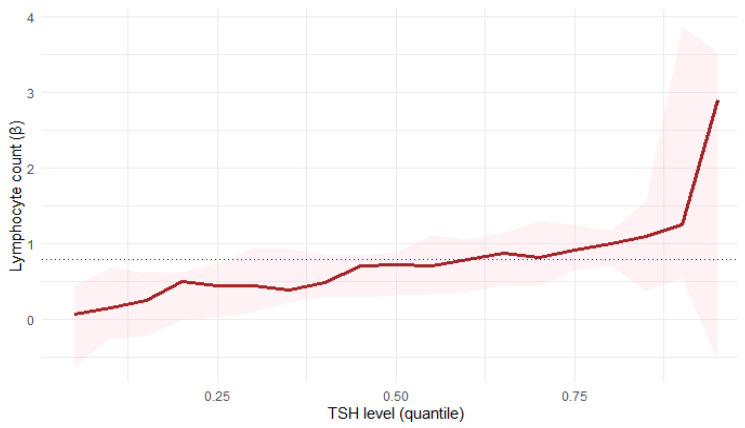
Quantile regression of the relationship of lymphocyte count and TSH.

Quantile regression confirmed a statistically significant relationship between TSH levels and lymphocyte count (Figure 8):

- At the 25th percentile (τ = 0.25), lymphocyte count showed a positive correlation with TSH levels (β = 0.4524, *p* = 0.0329), which indicates an increase of one unit in Lymphocyte count; there was 0.45-unit increase in TSH level on average.
- At the 50th percentile (τ = 0.5), lymphocyte count showed an even stronger association with TSH levels (β = 0.7279, *p* = 0.0009).
- At the 75th percentile (τ = 0.75), lymphocyte count showed the strongest correlation with TSH levels (β = 0.9211, *p* = 0.0021).

These findings suggest a stronger TSH influence on lymphocyte count in patients with higher lymphocyte levels. However, in the analysis, a clear increase in the variability of the estimates was observed for extreme τ values, which is typical of quantile regression and results from the limited number of observations. Therefore, the interpretation of the results should focus on the lower and middle quantile ranges, where estimates are more stable and representative.

In the female subgroup, a statistically significant positive association between lymphocyte count and TSH was observed, particularly in the middle quantile (τ = 0.5, *p* = 0.0017), with the effect persisting in higher quantiles (τ = 0.75). This indicates that for females, higher lymphocyte counts were associated with higher TSH levels.

For males, the relationship was weaker and not statistically significant across the analysed quantiles (τ = 0.25–0.75, *p* = 0.1388–0.2507). The regression slopes (β) remained positive but smaller than in women and characterised by greater uncertainty, suggesting a less stable association. A comparison of these results can be seen in Table 5.

**Figure 9:**
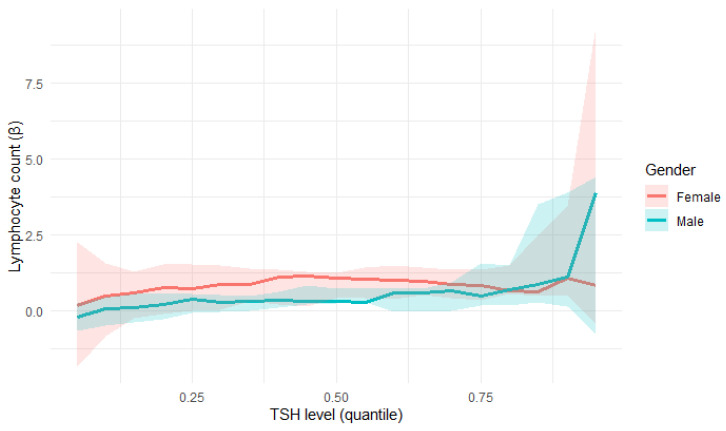
Quantile regression of the relationship of lymphocyte count and TSH (divided by gender).

The positive association between lymphocyte count and TSH level remained significant when the entire cohort was analysed, while the gender-stratified analyses, depicted in Figure 9, suggest that this effect was predominantly driven by the female subgroup. However, this result may also be partially influenced by the smaller sample size after stratification by gender.

## 4. Discussion

The results of our study indicate a significant relationship between TSH levels and selected inflammatory markers in patients with SARS-CoV-2 infection. These findings are consistent with previous reports of frequent thyroid dysfunction, including euthyroid sick syndrome (ESS), during SARS-CoV-2 infection, which has been found to correlate with disease severity [4,8,9,14]. These associations may be due to direct tissue injury caused by the virus via ACE2 receptors, as well as the secondary inflammatory response mediated by pro-inflammatory cytokines such as IL-1β, IL-6, TNF-α, and IFN-γ [3,4,8,9].

Furthermore, our findings may have practical implications for assessing the risk of severe disease. Previous studies have mainly focused on conventional inflammatory markers such as C-reactive protein (CRP) or procalcitonin, whose prognostic value, although useful, remains limited. In a study by Baldelli et al. [3], thyroid dysfunction was observed in patients without overt thyroid disease, suggesting a broader systemic response to infection. Similarly, Lui et al. [10] reported that lower TSH and fT3 concentrations were associated with more pronounced immune dysregulation and a more severe disease course. In this context, our findings, which show a correlation between TSH, lymphocyte count, and NLR, indicate that TSH could serve as an indirect marker of immune integrity. Therefore, decreased TSH levels may reflect immune exhaustion in critically ill patients [4,8,9].

It should also be noted that disturbances to the HPT axis during SARS-CoV-2 infection are not necessarily permanent. Lui et al. [10] found that thyroid function normalised within several weeks of recovery in many patients. However, Macedo et al. [7] demonstrated the presence of SARS-CoV-2 particles in thyroid follicular cells, indicating possible direct viral damage that could prolong dysfunction. Similarly, Duntas and Jonklaas [5] emphasised the bidirectional relationship between inflammation and thyroid regulation: systemic inflammation suppresses thyroid activity, while hormonal alterations may influence immune dynamics in turn. More recent studies have suggested that TSH may be an additional prognostic marker of the severity and outcome of SARS-CoV-2 infection [15].

Taken together, these findings support the potential utility of routine thyroid function monitoring, particularly during convalescence, as a complementary element of clinical assessment, especially for elderly individuals and women, who are more prone to HPT axis disturbances. Prospective studies evaluating TSH, fT3, fT4, and cytokine profiles, similar to that conducted by Duntas and Jonklaas [5], could further clarify the existence of a shared immuno-endocrine regulatory mechanism shaping the clinical course of SARS-CoV-2 infection [15].

In our study, lower TSH values were associated with reduced lymphocyte counts and abnormal platelet levels. Similar findings were reported by Lui et al., who demonstrated an independent association between TSH and free triiodothyronine (fT3) levels and lymphocyte counts. This suggests a link between the function of the hypothalamic–pituitary–thyroid axis and the immune status of patients. In another study by the same group, thyroid dysfunction was found to be significantly associated with the immune profile, clinical status, and outcomes of patients [10]. Baldelli et al. [3] emphasised that decreased fT3 and TSH levels are related to more severe forms of the disease and may serve as prognostic markers. Gong et al. [8] confirmed that low TSH levels in non-thyroidal illness syndrome are associated with an increased risk of adverse outcomes. Data from different populations, including Nigeria [14], suggest that these abnormalities are global rather than regional.

A reduction in TSH levels in severe cases of COVID-19 may represent an adaptive mechanism consistent with ESS, which aims to reduce the metabolic rate and conserve energy in situations of severe metabolic stress [4,8,9]. However, the persistence of such disturbances may negatively impact recovery, particularly in patients with comorbidities or pre-existing thyroid dysfunction.

The limitations of our study include its retrospective design and relatively small sample size, as well as the absence of long-term follow-up data and fT3 and fT4 measurements. Further prospective studies involving larger cohorts and follow-up assessments of thyroid function after recovery from SARS-CoV-2 infection are needed to evaluate the persistence and clinical relevance of these changes [15].

Our results also suggest that TSH has potential prognostic value. As it correlates with lymphocyte count, and NLR and TSH measurement is inexpensive and widely available, TSH could be considered an additional marker of severity of infection with SARS-CoV-2 alongside C-reactive protein (CRP), D-dimers, or procalcitonin. Another noteworthy finding is the sex-related difference: the positive correlation between TSH and platelet count in the female subgroup may reflect hormonal and immunological differences influenced by oestrogen, a phenomenon that requires further investigation.

One plausible hypothesis is that the hypothalamic–pituitary–thyroid (HPT) axis and the immune response share a regulatory pathway. Pro-inflammatory cytokines (IL-6 and TNF-α) have been shown to suppress TSH secretion and promote lymphopenia, suggesting a potential link between endocrine and immune dysregulation [9]. This observation is further supported by systematic evidence: Campi et al. [16] demonstrated in a meta-analysis that thyroid hormone alterations, including low TSH and fT3, are consistently associated with immune dysregulation and poorer clinical outcomes in patients with COVID-19. These findings emphasise the clinical relevance of monitoring thyroid function as part of a patient’s comprehensive assessment. Future studies should include fT3 and fT4 measurements, as well as longitudinal follow-up of patients after recovery, to determine the persistence of thyroid dysfunction. Incorporating more detailed immunological markers, such as cytokine profiling or lymphocyte subpopulation analysis, may further clarify the underlying mechanisms of these associations [15,16].

It is important to note that our analysis included a relatively small cohort of 105 patients, which limits the statistical power of our findings. Larger studies, such as those conducted by Lui et al. (191 patients) [10,11] and Gong et al. [8], have demonstrated a wider variety of associations between thyroid function and immunological parameters. Therefore, some of the non-significant results in our cohort (e.g., C-reactive protein (CRP), D-dimers, or procalcitonin) may reflect insufficient statistical power rather than a true absence of association. Moreover, as mentioned above, the number of records of each parameter was different due to lack of data. In this context, our results should be interpreted as supporting previous observations, but requiring confirmation in larger populations [14].

## 5. Conclusions

Our study suggests that TSH levels may be associated with lymphocyte count and NLR in patients with COVID-19, as well as with PLT in the female subgroup. These findings suggest that TSH could be used as an additional indicator when assessing patients’ conditions. TSH is a widely used indicator in thyroid dysfunction screening, but it is often overlooked. It is worth noting that TSH can be a helpful tool in evaluating a patient’s condition. However, it should not be used in isolation and should always be considered alongside clinical examination and, if possible, fT3 and fT4 levels. In contrast, other commonly used inflammatory markers, such as C-reactive protein (CRP), D-dimer, white blood cell (WBC) count, lymphocyte percentage, and procalcitonin, did not show statistically significant associations in the evaluated group. While these results support the potential role of TSH as a biomarker of disease severity in patients with SARS-CoV-2 infection, the lack of evaluation of fT3 and fT4 levels in our study highlights the need for more comprehensive research into this relationship. Further studies are needed to confirm this hypothesis and explore its relationship with other inflammation markers.

## Figures and Tables

**Table 1:** Non-thyroidal autoimmune diseases and number of excluded patients.

Autoimmune Disease	Number of Patients
Ulcerative colitis	2
Asthma	2
Diabetes mellitus type 1	3

**Table 2:** Number of records of each analysed parameter.

Parameter	Male Records	Female Records	Number of Records
TSH	56	49	105
CRP	56	49	105
PLT	56	44	100
D-dimers	42	32	74
Lymphocyte count	41	37	78
WBC	55	49	104
Procalcitonine	33	28	61
NLR	38	33	71

TSH—thyroid-stimulating hormone; CRP—C-reactive protein; PLT—platelet count; WBC—white blood cell count; NLR—neutrophil-to-lymphocyte ratio.

**Table 3:** Distribution of C-reactive protein (CRP) and white blood cell count (WBC).

CRP	Quantity of Records	WBC	Quantity of Records
<10 mg/L	29	2–4 × 10^3^/µL	11
10–100 mg/L	53	4–10 × 10^3^/µL	68
>100 mg/L	23	>10 × 10^3^/µL	25

**Table 4:** Distribution of D-dimers, platelet count (PLT), and procalcitonin.

D-Dimers	Quantity of Records	PLT	Quantity of Records	Procalcitonin	Quantity of Records
<500 µg/L	20	<200 × 10^3^/µL	38	≤0.5 ng/mL	55
≥500 µg/L	54	≥200 × 10^3^/µL	62	>0.5 ng/mL	6

**Table 5:** Statistical significance of the quantile regression between TSH level and lymphocyte count.

Quantile	Females (n = 37)	Males (n = 41)	All Patients (n = 78)
0.25	*p* = 0.0714	*p* = 0.1388	*p* = 0.0329
0.5	*p* = 0.0017	*p* = 0.0945	*p* = 0.0009
0.75	*p* = 0.0589	*p* = 0.2507	*p* = 0.0021

## Data Availability

The data presented in this study are available on reasonable request from the corresponding author. (Due to privacy and ethical restrictions, the raw patient data are not publicly available).
